# Unusually aggressive immature neo-intimal hyperplasia causing in-stent restenosis

**DOI:** 10.5830/CVJA-2017-024

**Published:** 2017

**Authors:** McCutcheon Keir, S Triantafyllis Andreas, Bennett Johan, Adriaenssens Tom

**Affiliations:** Department of Cardiovascular Medicine, University Hospitals Leuven, Leuven, Belgium; Department of Cardiovascular Medicine, University Hospitals Leuven, Leuven, Belgium; Department of Cardiovascular Medicine, University Hospitals Leuven, Leuven, Belgium; Department of Cardiovascular Medicine, University Hospitals Leuven, Leuven, Belgium

**Keywords:** in-stent restenosis, neo-intimal hyperplasia, optical coherence tomography

## Abstract

This image illustrates a very unusual pattern of early and aggressive immature neo-intimal hyperplasia in a 52-year-old man with unstable angina, two months after deployment of a drug-eluting stent in the proximal left anterior descending artery.

A 52-year old man was admitted with unstable angina two months after deployment of a drug-eluting stent (DES) in the proximal left anterior descending (LAD) artery. Five months prior to the current admission he had undergone percutaneous coronary intervention (PCI) with a DES to his proximal right, proximal circumflex and mid-LAD coronary arteries. The patient had no cardiovascular risk factors apart from a family history of premature coronary artery disease.

Coronary angiography demonstrated in-stent restenosisof the proximal LAD stent (Fig. 1A). Optical coherencetomography (OCT) demonstrated various tissue responses tostent implantation (Fig. 1B). High-signal, smooth muscle-richmature neo-intimal hyperplasia was present within the stent in themid-LAD (Fig. 1C; asterisk) whereas signal-poor homogeneousaggressive immature neo-intimal hyperplasia was present at thelevel of the proximal stent edge, causing sub-total occlusion (Fig. 1D; double arrowhead line), with tissue protrusion clearly visiblebelow the immature neo-intimal hyperplasia in certain frames(Fig. 1E; arrowheads). Proximal to the stent, an inhomogeneousedgevascular response was observed (Fig. 1F). The focalrestenosis in the proximal stent segment was treated with anotherDES and post-dilated with a non-compliant balloon with a goodangiographic result (not shown).

**Fig. 1:**
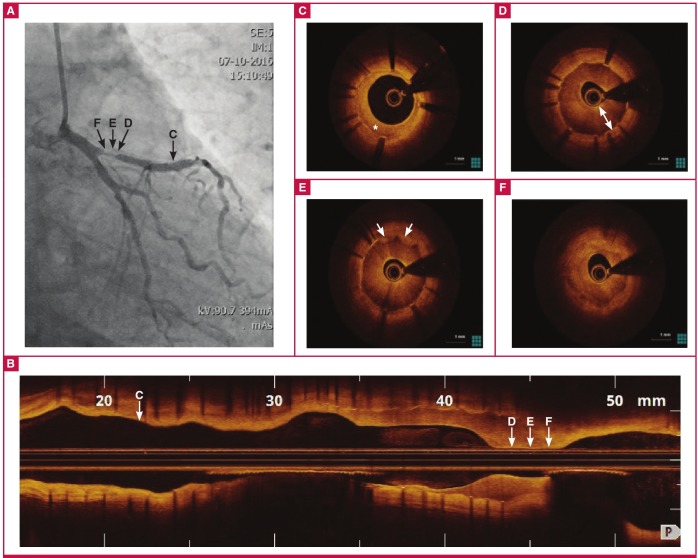
Fig. 1. A: Still frame of left coronary angiography showing restenosis in the proximal LAD. Letters in black correspond with the optical coherence tomography (OCT) images that follow. B–F: OCT from distal to proximal left anterior descending (LAD) and left mainstem demonstrating mature neo-intimal hyperplasia in mid-LAD stent (C, asterisk), aggressive immature neo-intimal hyperplasia in the proximal LAD stent (D, double arrowhead line) with tissue protrusion (E, arrowheads) and stent edge vascular response (F).

This image illustrates a very unusual pattern of early and aggressive immature neo-intimal hyperplasia. Although immature neo-intimal hyperplasia has been described,1 to our knowledge this is the first image of such aggressive immature neo-intimal hyperplasia.
